# Pericementitis

**Published:** 1888-12

**Authors:** F. S. Maxwell

**Affiliations:** Steubenville, O.


### Pericementitis.

By F. S. Maxwell. D.D.S., Steubenville, O.

[Read before the Ohio State Dental Society, Cincinnati, Oct. 16, 1888.]

Pericementitis is inflammation of the membrane associating the root of a tooth with its alveolus. The peridental membrane is a thick, fibrous membrane investing the roots of the teeth,—continuous with the periosteum of bone,—thicker than the periosteum of any part of the body, and increases in thickness with age. The peridental membrane is a reflection of the membrane that covers the alveolus, at the same time dipping down to line the pulp cavity, and is continuous with the mucous membrane covering the gum.

The peridental membrane,—the remains of the cemental pulp,—is called the "mother of the cementum," and the cementum being developed from the periosteum it therefore in after life receives its nourishment from it.

The peridental membrane consists of but one layer, in contra-distinction to the periosteum, which consists of two, the outer one, formed of connective tissue, the inner of fine elastic fibres, which is continuous with the peridental membrane, the outer layer disappearing and re-uniting with the inner after leaving the oral cavity. The fibres of the membrane are so arranged as to act as a cushion against a strain brought against the tooth, the arrangement being alike over the body of the root, but different at the apex, leaving a space between the apex and alveolus, called by Dr. Black the apical space.

These fibres again come together at the rim of the alveolus, forming a thick mass called the dental ligament, and are then continuous with the periosteum. The peridental membrane receives its blood supply from a branch of the artery supplying the pulp, and which passes into the apical space before dividing, as one source, and from another by the anastomosing of these vessels with similar ones supplying the gums. Thus you see the membrane has an arterial supply from two sources, and any injury affecting the supply from one will cause no stoppage with the other, and consequently the periosteum does not suffer. The nerve supply is the same.

The cementum of the root is supplied with nutrient material from the peridental membrane, hence if *some* of this blood and nerve supply is cut off, the cementum does not suffer for want of nourishment. One of the most important offices of the peridental membrane is its sense of touch.

The enamel of the tooth has no such sense and the pulp only conveys painful impressions, hence every touch upon the tooth *must* be reported by its peridental membrane. Its value is therefore apparent, but like every other part of the human body, it is subject to disease. Pericementitis *proper* has its seat in the apical space, following the death of the pulp, immediately preceding alveolar abscess, and is the beginning of the most painful affection to which the teeth are liable. It *seldom* occurs during the life of the pulp, being augmented by the escape of poisonous gases through the apical foramen and into the surrounding tissues. Pericementitis may exist under two forms, acute or apical pericementitis becoming chronic in character, and those cases having their origin at the gingival border, *some* of which are *constitutional* and chronic in form, the acute or apical form always occurring from a diseased pulp. By far the greater number of cases come under the acute form, and the most frequent of these is *apical* or *true pericementitis.* This always begins in the apical space and near the apical foramen. This inflamed tissue, which is richly supplied with nerves, becomes exquisitely painful and is prone to terminate in alveolar abscess. There is no particular time for inflammation to begin, sometimes it immediately follows the death of the pulp, often is indefinitely delayed, and then, again, may never occur. "It is safe to state, though, that no tooth with an empty pulp-chamber is safe from apical pericementitis."

The symptoms of the disease vary considerably, but it usually manifests itself by a dull, and sometimes throbbing, pain about the effected tooth. This is at first relieved by pressure upon the tooth, but as inflammation increases, pressure produces extreme pain, and inflammation elevates the tooth in its socket. The gum becomes red, changing to a purplish hue, the pain becomes continuous and more violent, and an abscess may be established in from one to five days.

It is not very difficult to diagnose a case of acute apical pericementitis. Pain caused by pressure upon the tooth is a symptom distinguishing it from hyperæmia. In diseases of the pulp the affected organ does not become tender to the touch until the inflammation has passed through the foramen, and the patient is uncertain as to exact location. In apical pericementitis the pain is definitely located and the tooth is *not* subject to thermal changes, the reverse in diseased pulp. Having, then, diagnosed our case, the first thing to do, to obtain relief, is to gain free access to the root canals and cleanse with suitable instruments. Then saturate the cavity, after having first applied the rubber-dam, with some good non-irritating disinfectant,—*not creosote*,—carbolic acid and iodoform will do. After thoroughly disinfecting, the pulp chamber should be loosely filled with cotton containing some antiseptic dressing, and the cavity sealed temporarily. Usually the pain subsides within three or four hours, but occasionally will continue, in event of which, counter-irritation is necessary, which can be best made by means of chloroform applied directly over the end of the root, or better still, by capsicum plasters; I use Dr. Frank Darby's. In all cases a brisk saline cathartic is beneficial. Heroic treatment, consisting of making an external opening over the apical space, is sometimes resorted to, but this is seldom necessary. Chronic apical pericementitis has all the characteristics of the acute in a modified form, excepting that the pain is intermittent. This form of disease is likely to be confounded with phagedenic pericementitis, but an examination of the peridental membrane will render a diagnosis clear.

Diseases of the peridental membrane having their beginning at the gingival, or gum margin, have been very fully described in past years. I was always taught to group them for description. Dr. Riggs, of Hartford, Conn., a few years since, called attention to the importance of knowledge in this particular branch, but nothing has been made clear until Dr. Black took hold of it. He styles these classes of disease of the peridonteum as gingival, calcic, and phagedenic pericementitis. The gingival are for the most part subject to the patients' general health and are therefore constitutional in character, being caused by poisonous substances circulating in the blood and having an affinity for the gingival organ. We have mercurial gingivitis and gingivitis caused by typhoid fever, scurvy and other skin diseases. Simple gingivitis is but the starting point of grave diseases, the swollen condition of the gums being a receptacle for calculus. Calcic pericementitis is caused by deposits of calculus on the necks of the teeth. It is certainly a serious trouble and causes, perhaps, the loss of as many teeth as caries.

There are two varieties of deposit, depending upon the location. One, the serumal, derived from the serum, and is always deposited *under* the gum; the other, the salivary, from the saliva, and is found on the necks of the teeth but *not beneath* the gum. The serumal deposit has a tendency to go to the end of the root, is in the form of a hard, brownish crust, or little nodules, and is not limited to the neighborhood of the salivary glands, as is the salivary calculus. When a slight deposit has taken place it becomes an irritant, and, though it may remain in position a considerable length of time before trouble ensues, it eventually destroys the peridental membrane. The most aggravating part of the disease is that it may go to the end of the root before its presence becomes known, the gum not receding with the alveolar wall and peridental membrane. The inflammation continues, pus flows from the sockets, the peridental membrane thickens about the end of the root, and although the tooth may be quite loose in its socket, it is still held firmly at the apex. This condition distinguishes it from phagedenic pericementitis, in which the membrane is destroyed at the *end*, but clings to the *sides* of the root. The teeth in this condition are, in reality, lost. The deposit of salivary calculus manifests itself in much the same manner as those just described, excepting that it is more pronounced in the neighborhood of the ducts of the salivary glands. As the deposit increases, the tendency is to encircle the tooth, and, going from one tooth to another, may spread over the entire arch. I have often seen cases where there were deposits of both the serumal and salivary variety, the serumal occupying the necks of the teeth *remote* from the salivary ducts. Salivary calculus is generally of a light yellow color, oftentimes dark, is much softer, deposited in larger quantities, and more irritating to the soft tissues than the serumal. Its effect upon the peridental membrane, however, is not so fatal as the serumal, unless completely encircling the tooth, when the tendency is to accumulate more rapidly, and the continuance of accumulation brings evil results. The peridental membrane, happily, when free from irritation, heals rapidly, and it is to this recuperation that makes the operations of replantation, transplantation, and implantation successful (?), providing the disease has not previously destroyed the peridental membrane. The treatment for calcic inflammation of the peridental membrane is a removal of the cause, and *thoroughness* in so doing, an operation sometimes very difficult to accomplish. The leaving of the smallest portion of deposit is but a flame to the fuel, and is as fatal as a portion of decay on the *margin* of a filling.

Suitable instruments are necessary, and in manipulating them the greatest care should be taken not to injure the gum, unless there should be a hypertrophy of that tissue. A speedy recovery should follow the removal of the calculus, unless the case is of long standing where there has been absorption of the alveolus and a thickening of the peridental membrane. In such cases I have always used a 30 per cent. solution of chloride of zinc *under* the free margin of the gums. Unless micro-organisms are present, escharotics should not be used in *after* treatment, but it is always well for a day or two to use carbolic acid in strong solution. If the gum returns to its normal condition, and the teeth are tight in their sockets, you can feel safe in predicting a cure, but not unless you have these conditions. Phagedenic pericementitis is a disease of the peridental membrane. The inflammation is of that peculiar character whereby the death of the membrane precedes that of the alveolus. The first sign of the disease is at the gingival margin when the gums become red, but without inflammation, which redness somewhat disappears as the disease advances toward the peridental membrane. The fibres of the latter seem to break down, or meet, under the exciting trouble, and a thin-bladed instrument can readily be passed along the root farther than it should.

Pus forms in *pockets* about the root and the tendency is to progress toward the apex and eventually destroy the entire root membrane. Neighboring teeth are generally attacked. Unlike serumal pericementitis, the *end* of the *root* may be entirely stripped, when the disease is not accompanied by calculus deposits, and the tooth still held in its socket by its membrane on the *sides* of the root where it may be but little affected. The alveolar wall is *afterwards* absorbed (Black), that is, in proportion to the advancement of the diseased membrane. The roots may be absorbed (Magitot) in connection with the disease, but the cases seem rare. Calculus deposits complicate the trouble, and a majority of cases have such deposits, but its absence does not make the case any the less destructive. In serumal calculus, with nodules under the gum, unless very careful as to cleanliness, salivary calculus will form also. "When this occurs in considerably quantity the appearance of disease becomes much more apparent because of the greater inflammation of the gum tissue caused by the calculus." Here we have, in fact, two diseases existing together: calcic inflammation from deposits of calculus, and phagedenic inflammation further up toward the apex. It is evidently this dual form, so often presented by these affections, that has so long delayed the recognition of the phagedenic variety as an independent disease. Great difference exists in regard to healing after removing the calculus, although the performance of some may be perfect.

Our results will be more satisfactory if our diagnosis is accurate. In phagedenic pericementitis *pockets, as a rule,* are found along the roots. The peridental membrane is destroyed much further than the calculus has extended on the root, "and in the final loosening of the tooth it will be found to have been held by a portion of membrane on the sides, the membrane at the apex having been destroyed." These differences afford pretty reliable ground for diagnosis. Sometimes, though, we find cases where pus is burrowing from under the gum and we are led to believe we have a case of alveolar abscess. In event of this we can look for other neighboring points of attack in order to diagnose our disease. According to Dr. Black, the etiology of phagedenic pericementitis seems to be from the fungus of some micro-organism from the fact that medicines that destroy micro-organisms produce favorable results here. It is observed that the disease will not flourish except in locations in which a fungus growth would have some form of protection against the free flow of the buccal fluids, such as the pockets, formed at the gum margin, will give, hence the cure of the disease by the removal of all free margins of the gums. This disease is purely *local*, and not dependent upon any poison circulating in the blood, or of any systemic disorders, and is not hereditary, as the deposit of calculus may be, nor do the deposits of calculus favor its development. Some writers regard it as constitutional, but if it is, it is confined to scrofulous, gouty, or rheumatic persons, with anæmia as the predisposing cause. The treatment of phagedenic pericementitis calls for certain operations in common with the treatment of calcic inflammations, the first thing in every case being the removal of all deposits from the neck and roots of the tooth, such deposits being generally situated far up under the gum. This calculus of the serumal variety must be *thoroughly* removed, and what operation of the oral cavity, to be successful, should not be thorough, as the smallest particle remaining acts as an irritant. Phagedenic, unlike calcic inflammation, does not end with the removal of the calculus. Where there is a rapid destruction of the tissues, including the peridental membrane and the alveolus, it is better to cut away with a sharp excavator sufficient to obtain solid or healthy surface, or until the bone is felt to be firm and resistant. In this case, as in all others, great care should be taken not to injure the gingival margin, the injury sustained further toward the apex is of no importance, comparatively, but if this gum margin is injured sufficiently to cause a slough, our efforts to heal the peridental membrane will be defeated. After the operation, the gum should lie closer to the neck of the tooth than before beginning, acting as a barrier to the saliva, and exclusion of micro-organisms. If there are no thickened or roughened margins to interfere with the contact of the parts, medication may begin at once. The pocket should be thoroughly washed with peroxide of hydrogen, as an antiseptic, after first removing the debris, and a little bichloride of mercury may be added if so desired. These can best be injected by a Dunn syringe, and the treatment continued with chloride of zinc, as an astringent, for a time or two. Follow this with an application of oil of cinnamon, two parts to one of carbolic acid, as a stimulent, to be repeated once every three or four days. Dr. Harlan recommends a solution of iodoform and eucalyptus, or iodoform and cinnamon, either of which is a good stimulant and antiseptic, and should be packed into the pockets, not injected. In the after treatment, care should be taken not to injure the granulations, the parts do not heal by first intention, and the mouth rinsed frequently with a slightly diluted listerine wash. If our treatment has been successful, an attachment will take place between the cementum and peridental membrane, even to a dead tooth, for this fact was demonstrated by Hunter in a case of replanting in 1778. "In the treatment of phagedenic pericementitis the complete reformation of the peridental membrane should be expected if the gingival margin be left intact." Just so far as the margin of the gum may have receded from its normal position, just so far will we fail in reformation of the peridental membrane; that is, if a portion of the root is left uncovered, there cannot be a reproduction of the membrane at that point; however, the peridental membrane may be there with no reproduction of the alveolar wall. Some operators resort to the sponge-graft for the rebuilding of the peridental membrane, but I regard it as a dangerous practice, at least, until I hear of more success in its use.

DISCUSSION.

*Dr. Taft*—This paper impresses me as being a very good one. There is but little, if anything, in the paper that I would criticise especially. This may be said, however, that the writer did not classify the different stages of this affection as closely as he might have done. But he certainly presented a very good resume of the subject, and a good presentation of the varieties of the diseases that arise from this tissue. His mode of treatment also is good. There are, of course, other modes of treatment from which very good results have been obtained.

It is important in treating periostitis, to note all irritants, and the influence that each may have on the diseased tissue. There is, perhaps, none requiring more special attention than salivary calculus. This, in many instances, occasions so much irritation that restoration cannot be obtained while it is present.

His remarks about the removal of all foreign substances are good. The removal of diseased or dead bone, and even soft tissue, is sometimes a necessity if the best results are to be attained.

*Dr. Smith*—I do not rise to discuss the question, but simply wish to state that there is one point, if I am correct, in the paper that I can not quite agree with, and that is that we do not have pericementitis without the death of the pulp. The pulp becomes inflamed, and it is possible we may have pericementitis. When the pulp returns to its normal state, the symptoms of pericementitis subside.

*Dr. Maxwell*—I think Dr. Smith misunderstood me, for that comes, as I said, from the poison escaping from the sloughing pulp which causes the inflammation of the tissue. I do not think that you have ever found that sort of inflammation without gas being present.

*Dr. Custer*—There is one point that has not been touched upon, and that is, that pericementitis is not always the result of a dead pulp. In my limited practice I have found that in nearly every case where there is pain there is a live pulp instead of a dead one. Now, if there is a continuity of tissue, that pathological condition will spread and it will produce inflammation of the membrane in the apical territory. I would like the opinion of others on this point.

*Dr. Taft*—I think Dr. Custer in his remarks mistakes in one respect; and that is in which the disease would take place at the end of the root. It is true that occasionally with a live pulp there will be inflammation of the periosteum, but it is not often—at least I have not found it so—and it is not always when the disease occurs that it is found at the end of the root. In almost every instance when there is inflammation at the end of the root, the pulp is dead. I do not now remember to have seen a single instance in which that affection was found at the end of the root, that the pulp was not dead. But this affection may start from a diseased gum by violence upon a tooth, and then it involves all of the membrane covering the root. It may extend to the end of the root, but in such a case it will be no more severe there than elsewhere, except possibly in a certain kind of violence. A blow upon the end of a tooth may, of course, bruise the membrane at the end of the root more than elsewhere, and inflammation arise. And such inflammation may be more marked there than elsewhere. Only local treatment in the main was referred to, but little was said on general treatment, and perhaps we do not enough appreciate the importance of general treatment, the local being that generally employed, and nearly all cases require local treatment. In many cases quinine will operate very well, giving prompt relief.

*Dr. Bell*—As I understand it pericementitis is simply an inflammation of the periosteum, and that there are a great many ways by which that trouble is brought about. It is brought about more often by mechanical means than by the poisonous material escaping from the pulp. In one instance this trouble was brought about by holding a pencil. The trouble embraced four of the superior and inferior teeth. It has been brought about by carelessness on the part of the operator allowing a filling to remain unfinished. Systemic treatment for the trouble I do not believe is necessary except in very extreme cases. A great many times the cause is mistaken and teeth destroyed when it is entirely unnecessary.

